# Towards an Optimized Fetal DHA Accretion: Differences on Maternal DHA Supplementation Using Phospholipids vs. Triglycerides during Pregnancy in Different Models

**DOI:** 10.3390/nu13020511

**Published:** 2021-02-04

**Authors:** Antonio Gázquez, Elvira Larqué

**Affiliations:** 1 Department of Physiology, University of Murcia, 30100 Murcia, Spain; antonio.gazquez@um.es; 2 Biomedical Research Institute of Murcia (IMIB-Arrixaca), 30120 Murcia, Spain

**Keywords:** docosahexaenoic acid, pregnancy, supplementation, egg yolk, microalgae, placenta

## Abstract

Docosahexaenoic acid (DHA) supplementation during pregnancy has been recommended by several health organizations due to its role in neural, visual, and cognitive development. There are several fat sources available on the market for the manufacture of these dietary supplements with DHA. These fat sources differ in the lipid structure in which DHA is esterified, mainly phospholipids (PL) and triglycerides (TG) molecules. The supplementation of DHA in the form of PL or TG during pregnancy can lead to controversial results depending on the animal model, physiological status and the fat sources utilized. The intestinal digestion, placental uptake, and fetal accretion of DHA may vary depending on the lipid source of DHA ingested by the mother. The form of DHA used in maternal supplementation that would provide an optimal DHA accretion for fetal brain development, based on the available data obtained most of them from different animal models, indicates no consistent differences in fetal accretion when DHA is provided as TG or PL. Other related lipid species are under evaluation, e.g., lyso-phospholipids, with promising results to improve DHA bioavailability although more studies are needed. In this review, the evidence on DHA bioavailability and accumulation in both maternal and fetal tissues after the administration of DHA supplementation during pregnancy in the form of PL or TG in different models is summarized.

## 1. Introduction

There is a growing interest in the effects of maternal diet consumed during pregnancy on both development and fetal programming of many physiological functions. During pregnancy and lactation there is an elevated docosahexaenoic acid (22:6 omega-3, DHA) requirement in the fetus and neonate as it is a critical building block of brain and retina [1,2,3]. In the last trimester of pregnancy, it is estimated a fetal accretion of 67 mg of omega-3 fatty acids (FA) per day, mainly DHA, and around 5% is delivered to the brain (3.1 mg/d) [4,5]. DHA conversion efficiency from α-linolenic acid (18:3 omega-3), its essential FA precursor, is very low (<1%) in fetus, placenta and newborns [6,7,8,9], being therefore insufficient to satisfy the high supply of DHA needed by the growing fetus [10,11]. Moreover, several studies have shown that supplementation with α-linolenic acid in human adults is not a good strategy to increase DHA levels, being necessary the direct supplementation with the preformed DHA molecule to observe: enhanced DHA status in blood and tissues [12], higher transfer of DHA to the fetus [13] or even to increase DHA secretion in human milk [14].

## 2. DHA Recommendations and Health Outcomes

### 2.1. DHA Intake during the Perinatal Period

**Figure 1:**
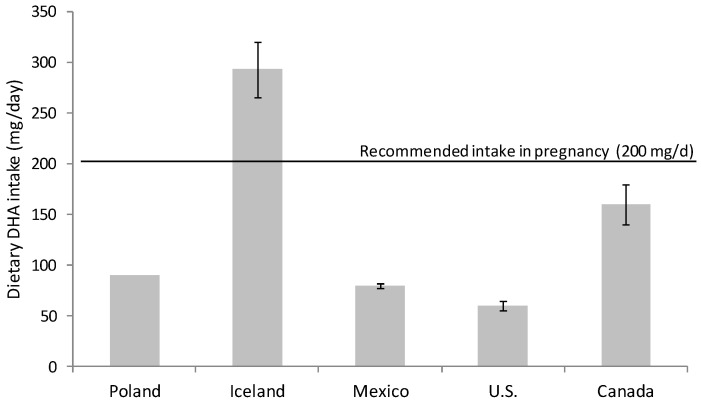
Estimated dietary docosahexaenoic acid (DHA) intake in pregnant women from different countries. Data from Innis and Elias 2003 [21], Parra-Cabrera et al. 2011 [18], Gunnarsdóttir et al. 2016 [23], Wierzejska et al. 2018 [24] and Zhang et al. 2018 [22].

Omega-3 FA intake had fallen during the 20th century; the development of the modern vegetable oil industry, the use of cereal grains and the change in eating habits have produced a remarkable disparity in the ratio of consumption of omega-6 and omega-3 FA [15,16]. Omega-6 FA consumption, mainly in the form of linoleic acid (18:2 n-6), has increased at the expense of omega-3 FA (DHA and eicosapentaenoic acid (EPA, 20:5 n-3)) in the general population [15,16,17]. Nowadays, the intake of DHA in developed countries with free access to food of animal origin rich in micronutrients and omega-3 FA is highly variable. There are several studies evaluating DHA consumption that warn about inadequate dietary DHA intake for many women during pregnancy [18,19,20] (Figure 1). It is especially alarming the situation in western countries, for example in Canada and U.S. where DHA intakes are especially low, revealing that a majority of childbearing-age and pregnant women consume less than the recommended DHA dose [21,22] (Figure 1).

The preferential placental uptake and transfer of DHA to the fetus in relation to other FA (palmitic, oleic and linoleic acid) has been demonstrated by the administration of stable isotope-labelled FAs to pregnant women [25,26]. Moreover, the percentage of DHA and arachidonic acid (AA; 20:4 omega-6,) both in plasma and adipose tissue is higher in the neonate than in the mother, which reveals the important role of the placenta in the concentration of these FA in the fetal compartment [27,28]. This process is known as “biomagnification” and is defined as selective enrichment of these FA in fetal, with respect to maternal plasma [29]. AA and DHA concentration in non-esterified FA (NEFA) of the intervillous space of the placenta is 3–4 times higher than in maternal blood outside the placenta [30]. This fact implies that there is certain selectivity of placental tissue for the release of these long-chain polyunsaturated FA (LC-PUFA) from the circulating lipoproteins.

It is well known that the maternal DHA intake, and hence maternal DHA levels, during pregnancy determines the DHA status of the newborn at birth and for several weeks following delivery [31,32,33,34]. Large observational studies have shown that women with low seafood intakes during pregnancy are prone to an increased risk of poor infant cognition and behavioral outcome [35,36]. Low levels of DHA and AA in maternal plasma and cord blood has been related to lower head circumference, lower birthweight, lower placental weight [32], and less cognitive and visual maturation during childhood [37,38]. Other studies found associations between omega-3 FA intake during pregnancy and lower risks of intrauterine growth restriction, preterm birth, allergies, and asthma in children [19,39,40]. However, some randomized controlled trials and meta-analysis reported inconsistent evidences and very few differences between child born from omega-3 supplemented vs. placebo mothers on long-term vision, growth and neurodevelopment outcomes [41,42,43,44,45]. Further follow-up studies are needed to assess the longer-term consequences and health outcomes for both mother and child of maternal omega-3 supplementation.

### 2.2. Dietary Recommendation during Pregnancy and Lactation

DHA dietary supplementation has been recommended by several health organizations [46,47]. European and global guidelines recommends the intake of at least 200 mg/d DHA during these periods, which can be met with two servings of fish per week [48,49,50]. The highest concentration of DHA is found in seafood, especially in oily fish (tuna, salmon, herring, mackerel, etc.) [51]. Smaller fishes are highly recommendable since they contain lower levels of methyl mercury and other contaminants than large-size predators [49]. Probably, the dose should be higher to detect significant effects on some outcomes but, due to the high variability in DHA intake from other sources, these recommendations are highly conservative.

DHA supplementation should be considered only if dietary consumption (natural sources) is not sufficient to meet the recommendations or when it is problematic due to food availability, socio-cultural dietary preferences, fish aversion, ethics issues (e.g., vegans), or other factors [52].

## 3. Lipid Sources Utilized in DHA Supplementation

The incorporation of LC-PUFA like DHA into dietary supplement products presents some technological problems because LC-PUFA are highly oxidable molecules; the large number of double bonds in their hydrocarbon chain makes advisable the addition of antioxidants or stabilizers to avoid FA oxidation. The development of microencapsulated products also allows adequate protection against oxidation, as well as the addition of these FA to powdered products [53,54]. Obtaining new fat sources with different properties, more economically competitive, healthier, or with greater bioavailability, has been an important part of the panorama of nutritional supplements for pregnant women. There are several fat sources that can be used for the manufacturing of DHA supplements. However, the lipid structure in which DHA is packaged may vary depending on the source utilized and sometimes, also on the production procedure utilized: phospholipids (PL), lyso-phospholipids (Lyso-PL), triglycerides (TG), monoglycerides, ethyl esters, etc.

### 3.1. Fish Oil

Fish oils are a good source of LC-PUFA omega-3 because they naturally contain high concentrations of both EPA and DHA, reaching up to 18–30% EPA + DHA in the form of TG [55]. The problems of sustainability of the large fish farms necessary for the production of these oils, typical fish odor that persists after deodorization processes and the constant increase in vegetarians and vegans contributed to the active search of alternatives for the production of this type of compounds [55,56]. In addition, the presence of some environmental contaminants in fish (e.g., methyl mercury, dioxins, and polychlorinated biphenyls), that accumulate along the marine food chain, being particularly concentrated in large predator species like shark, swordfish, or kingfish, are of special concern for pregnant women [57]. Methyl mercury is neurotoxic for the central nervous system of the fetus or newborn and its detrimental effects on neural function have been proved until young adult age [57,58]. Despite all of that, fish oil is still an important source of DHA for the production of many supplements not only for pregnant women but also for the general population.

### 3.2. Microalgae Oil

One of the most widespread alternatives to fish oil today is the oil obtained from culture in biofactories of different species of microalgae [56]. In fact, microalgae are the primary producers and the responsible of including LC-PUFA omega-3 in the seawater food chain [59]. Fish does not synthesize large amounts of LC-PUFA omega-3 but consumes microalgae rich in EPA and DHA or other organism fed with these microalgae [56]. Microalgae oil concentrates reach very high concentrations of DHA, containing up to 50–60% DHA in the form of TG with low levels of EPA, and have been tested in numerous investigations without any observed side effects in both animals and humans [55,60,61]. In fact, these oils are widely used in the food industry for the production of dietary supplements and the enrichment of several products [54].

### 3.3. Enriched Eggs

Chicken eggs are rich in protein and fat but have very little content of LC-PUFA omega-3 [62]. However, omega-3 enriched eggs can be produced by the addition of fish meal, flaxseed oil or fish oil to hen diet [54,63]; omega-3 FA increase significantly in fortified eggs resulting in up to 180 mg DHA/egg, which represent approximately the daily DHA recommended intake [64,65]. In contrast to fish and microalgae oils, egg yolk FA are distributed not only in TG but also in PL molecules (~30% of total lipids), being DHA esterified almost exclusively in phosphatidylcholine (PC) structures [66]. It has been demonstrated that consumption of omega-3 fortified eggs enhances LC-PUFA omega-3 status, including DHA levels, in breast and formula-fed infants [67] and healthy adult subjects [68,69].

Egg yolk is one of the most important sources of dietary PL of animal origin [70]. A typical Western diet contains about 3–6 g/d PL (4–8% of total fat) and a large egg can provide up to 0.8 g PL [71,72]. PC is the predominant PL species accounting for approximately 72% of the total egg PL; other PL are present in lesser quantities: 20% phosphatidylethanolamine (PE), 3% lyso-phosphatidylcholine (Lyso-PC), 3% sphingomyelin, and 2% phosphatidylinositol [70]. Beneficial health effects of dietary PL have been described over the last years related to cholesterol absorption, blood lipid profiles and cardiovascular disease risk, reduction in inflammatory processes, improvement of immunological functions, neurological development and disorders, anti-cancer properties, etc., [73,74]. However, more research is needed in order to discern what are the mechanisms involved and which effects are due to PL structure and which to the effects of FA or FA-derived metabolites carried in dietary PL [74].

### 3.4. Krill Oil

Krill are shrimp-like small crustaceans that live in the Antarctic ocean. Euphasia superba is the predominant species, known as Antarctic krill, and the main source of extracted krill oil [75]. Krill oil contains a considerable amount of DHA (~15%) bound to PL structures, primarily in the form of PC [75,76]. Like fish, marine microalgae are the source of LC-PUFA for krill [59]. However, in contrast to large fish, krill have a short lifespan (1–2 years) and, because they live in clean waters, are free of heavy metals, pesticides, and dioxins [77]. In the last few years, there has been a remarkable increase in the research of krill and krill oil for its health benefits in hyperlipidemia, chronic inflammation, arthritis, and premenstrual syndrome complications [75].

### 3.5. Lyso-Phospholipids

Lyso-PL, especially Lyso-PC, has shown to be a preferred physiological carrier of DHA to the brain and retina in some studies, being more efficiently taken than the NEFA, PL or TG form [78,79,80,81,82]. A similar observation has been made for erythrocytes, where DHA Lyso-PC is the major source of DHA for these cells rather than NEFA [83]. In addition, some authors have suggested that maternal erythrocytes may be a potential reserve of LC-PUFA and a preferred vehicle of them to the placenta [84]. Thus, Lyso-PL might represent an additional source of FA for the placenta. Table 1 summarizes the most relevant in vivo studies reported on Lyso-PL DHA bioavailability.

It has been demonstrated that Lyso-PL unsaturated FA at sn-2 position easily migrates to the sn-1 position in physiological conditions due to the higher reactivity of its primary alcohol [91,92]. 1-acyl Lyso-PL can be hydrolyzed by phospholipase A1, being its metabolic fate uncertain while 2-acyl is quickly reacylated and maintained in a PL structure [91]. On the other hand, during gut digestion sn-2 FA is hydrolyzed by pancreatic phospholipase A2 and that implies a lower retention in the PL structure [93]. The position of DHA within the PL molecule can affect its tissue accretion, differential incorporation of sn-1 and sn-2 Lyso-PC DHA has been reported in different brain regions in adult mice [81]. A structured 2-acyl Lyso-PL for DHA with the sn-1 position blocked (addition of an acetyl group, AceDoPC) has been synthesized in order to prevent the migration of DHA from sn-2 to sn-1 position [94]. Considering that 2-acyl Lyso-PC is the physiological form, this new molecule of DHA Lyso-PC may represent a more stable and bioavailable source of DHA for the brain and the placenta. Till now, oral intake of AceDoPC has shown good in vivo incorporation to human red blood cells PL [90,95]; additionally, it was more rapidly accumulated in brain of 20-day-old rats compared to the administration of DHA as NEFA while both compounds showed similar accretion in other tissues like plasma, heart or liver [86]. However, AceDoPC has been no tested in pregnant animals yet.

There is only one study evaluating the bioavailability of Lyso-PL form of DHA during pregnancy and it showed positive effects of maternal supplementation with DHA as Lyso-PC (obtained from egg yolk PL) in pregnant rats [85]. The authors found higher DHA content in the cerebellum and hippocampus, as well as a better score of learning and memory of pups at two months delivered by mothers supplemented with DHA Lyso-PC compared to DHA monoacylglycerides [85]. Based on available data, Lyso-PL forms of DHA might have a higher bioavailability than other lipid structures like PL, TG, or NEFA (Table 1). However, the evidences are still limited and more studies are needed to understand the biological consequences and metabolism of DHA supplementation as Lyso-PL, especially during critical periods of development like pregnancy.

### 3.6. Other Sources

#### 3.6.1. Animal Products

The search for new alternative lipid sources more efficient and cost-effective for improving the intake of DHA is an area of intense research. Enriching animal products (meat, meat products, and dairy-derived food) through diet fortification with vegetable and fish sources of omega-3 FA has shown promising results in DHA concentration of edible tissues and milk. However, the inclusion of these LC-PUFA in meat presents some technological problems related to oxidative stability, off-flavors and increased production of trans and conjugated FA in the ruminants [96,97,98,99].

#### 3.6.2. Plants

The increase in omega-3 LC-PUFA production and accumulation in plants by genetic engineering is also of interest. Plants are primary producers of essential FA (linoleic and α-linolenic acid) but lack of the natural capacity to synthesize LC-PUFA (AA, EPA, and DHA) [59]. The goal is to produce transgenic plants capable of accumulate omega-3 LC-PUFA to levels similar to that found in fish oil through the promotion of desaturase (Δ-5 and Δ-6 desaturase) and elongase (Δ6-elongase) activities [100]. Although this alternative seems very promising and may lead to significant production of omega-3 LC-PUFA for human consumption in the future, the levels of omega-3 obtained are much lower than the other types of lipid sources, the process is still expensive and the cultivation of these plants must go through a rigorous process of regulatory approvals [54,59,100].

## 4. Materno-Fetal Bioavailability of Different DHA Sources

The production of dietary DHA supplements has been a field of intense study and evolution in recent years. However, the use of different fat sources of DHA mean that we are taking this FA in different lipid structures, which may affect both intestinal digestion and absorption, lipoprotein distribution, metabolic fate, placental uptake and delivery to fetus [101,102,103]. All these observations open the door to thinking about what is the quantitative contribution of the different lipid fractions (NEFA, TG, PL, and cholesterol esters (CE)) of maternal plasma to the placenta and whether they differ in bioavailability. The incorporation of dietary FA into maternal circulating lipoproteins (mainly in the form of PL or TG) might be modified by the lipid form consumed in the diet. Therefore, it is interesting to study whether the consumption of DHA in the form of PL vs. the classical form of TG could lead to a more favorable plasma conditions for the placenta, enhancing the production of DHA-rich Lyso-PL and an increased placental uptake and fetal delivery of DHA. This would allow the design of new nutritional supplements for pregnant women with sources of DHA with higher bioavailability for the placenta. A summary of the main data from studies evaluating DHA bioavailability after TG or PL supplementation is reported in Table 2.

### 4.1. Intestinal Digestion and Absorption

**Figure 2:**
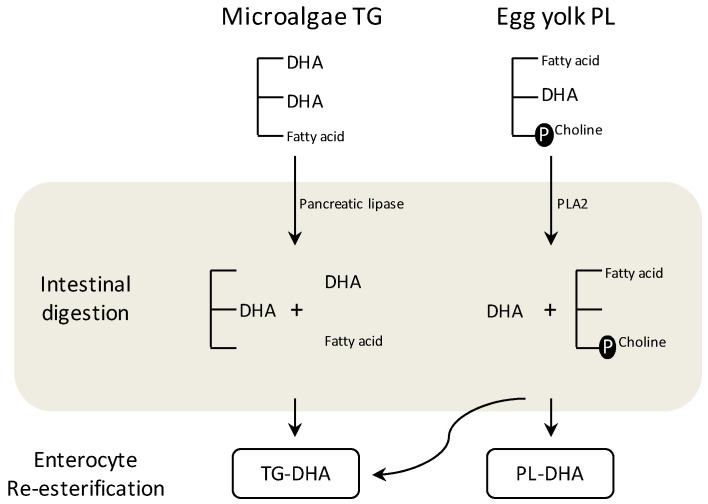
Intestinal digestion of dietary docosahexaenoic acid (DHA) ingested as phospholipids (PL) from egg yolk or triglycerides (TG) from microalgae oil schematic. PLA2, phospholipase A2.

The digestion of dietary fats and the subsequent FA absorption and assembly in plasma lipoproteins depends on the chemical structure in which they have been ingested (mainly TG or PL). TG digestion takes place in the small intestine where the FA of sn-1 and sn-3 positions are hydrolyzed by pancreatic lipase releasing the corresponding NEFA and 2-monoacylglycerol (Figure 2). A small part of the 2-monoacylglycerol is fully degraded to NEFA and glycerol. On the other hand, dietary PL digestion (mainly PC) occurs through the action of the pancreatic phospholipase A2 which releases the FA located at the sn-2 position generating a NEFA and a Lyso-PL, although a small part is completely hydrolyzed to NEFA and glycerol phosphocholine (Figure 2) [117,118].

The products generated during the digestion are captured by the enterocytes in a process not entirely established in which both passive diffusion processes and facilitated transport by FA binding protein associated with the plasma membrane (FABPpm), fatty acid translocase (FAT/CD36) and fatty acid transport protein (FATP) take place [101,119,120]. Once inside the enterocyte, TG and PL molecules are re-esterified and the NEFA can be incorporated into the same structure as they were part or into others that are being formed at the same time in the cell (TG, PL, or CE) (Figure 2). PL and TG captured or re-synthesized by the enterocytes are transported in the bloodstream in lipoproteins, mainly chylomicrons (QM, apolipoprotein B48) and very low density lipoproteins (VLDL, apolipoprotein B100), especially in fasting situations [121,122]. The ingestion of PL or TG modifies the diameter of secreted lipoproteins. PL ingestion produce lipoprotein with lower diameter, called by some authors “small QM”, than those secreted after TG ingestion [122,123]. However, the lipid source of DHA not only affects the dimension of lipoproteins secreted but also their FA composition. The four-week administration of LC-PUFA (AA and DHA) from fungal and tuna oil resulted in a preferential incorporation of these FA in PL fraction of low density lipoprotein (LDL) particles in neonatal piglets, while when egg yolk PL were used as DHA source (DHA in the form of PL) a higher incorporation in high density lipoprotein (HDL) PL was found [109]. This has been observed not only for LC-PUFA but in general for PC which after being absorbed is preferably incorporated into HDL lipoproteins [124], probably directly in the enterocyte and without liver intervention [125].

There is controversy on the effects of PL addition to the diet on FA intestinal absorption. Several studies revealed better fat digestion and increased FA absorption when egg yolk PL were added to diets of experimental animals or infant formulas [105,107,114,126]. In fact, Carnielli et al. reported that DHA from egg PL was better absorbed in premature babies than DHA from breast milk or algae oil (TG form) [114]. However, other authors showed better absorption of FA after TG sources administration than when using PL sources (egg yolk and krill oil) in neonatal piglets and rats [104,110]. Differences in composition or the presence of other lipid components in important amounts may also affect lipid digestion and FA absorption. For example, Amate and colleagues showed higher absorption of diet supplemented with egg yolk PL compared to egg yolk TG and lower absorption of pig brain concentrate PL compared to tuna and microalgae oil [105]. The authors argued that the presence of other lipid compounds such as cerebrosides, gangliosides, esphingolipids and Lyso-PC might have affected the intestinal absorption of the experimental fats [105].

Similarly krill oil, with DHA mainly in PL, has received criticism from some experts in comparative studies of LC-PUFA omega-3 bioavailability due to its changes in composition depending on the technological processing and the season of capture [127,128]. For example, it has been described that krill oil PL content ranged from 19 to 81% and that it can contain a high amount of DHA in lipid structures different to PL such as NEFA (up to 21% DHA) and FA ethyl esters, which clearly influences both FA absorption and bioavailability [76,129,130,131]. Other aspects such as intestinal maturity, metabolic differences inter-species, and microflora composition influence both fat digestibility and absorption processes making difficult the comparison of FA bioavailability studies [132].

Some authors have studied the positions of the lipid structures whose FA are most likely to be preserved after the process of intestinal digestion and absorption. It has been shown that FA esterified in the sn-2 position of the TG molecule is widely conserved in TG fraction of QM particles [133,134]. On the other hand, in the case of PL, the FA esterified in the sn-1 position of PC is also protected from digestive hydrolysis [117]. Therefore, the position that occupies the DHA within the molecule is important, it could determine in which structure this DHA will later appear in the bloodstream and enhanced or minimized the expected effects of dietary supplementation.

Egg DHA shows a high stereospecificity, being almost exclusively esterified in the sn-2 position of PC and PE molecules [66,135]. This implies that a large part of the DHA from egg yolk enters the enterocyte as NEFA and not in the form of Lyso-PC and may not be maintained in a structure of PL when moving to plasma (Figure 1). In pig brain concentrate PL something similar occurs, although the composition in terms of the type of PL is not the same. Egg PL are mainly composed of PC (87% PC and 11% PE) while pig brain concentrate by PE (44% PE and 24% PC) [135]. The effects that the type of PL might have on the digestion and absorption of FA are unknown. On the other hand, the distribution of DHA within TG molecules is also not the same in all sources; tuna oil contains about 50% DHA esterified in the sn-2 position of the glycerol [135], similar values have been also described for some microalgae oils [136]. However, in other sources, such as egg yolk TG, a similar distribution has been observed between the sn-1 and sn-2 position [66]. Artificial re-esterification processes in oil contribute to a random distribution of DHA between the three available positions of glycerol [128]. Little is known about the likely influence of the characteristics of each source and the composition of the rest of FA (saturated, monounsaturated, omega-6, etc.) on the digestion and absorption of DHA. However, DHA in the sn-2 position of TG has been shown to be better absorbed than those in sn-1 or sn-3 position [137,138].

Therefore, it is more likely that DHA from microalgae oil or fish oil TG (sn-2 position TG) remains on the same TG molecule structure than DHA from egg yolk (sn-2 position PL) (Figure 2).

### 4.2. Circulating DHA and Metabolic Fate

The lipid fraction in which administered DHA appears in blood circulation is important since it may determine in some grade the metabolic fate and metabolism, and hence the efficiency, of the DHA supplementation applied. It is not clear whether intake of DHA during pregnancy as PL form can be a better source with higher placental bioavailability and fetal brain accretion than the consumption of DHA as TG. The use of lipid sources with PL to evaluate the bioavailability of DHA for different organs in comparison with the administration of TG sources has been extensively studied in non-pregnant humans and animals.

Studies in full-term infants, children and piglets indicated that the plasma lipid fraction in which DHA is incorporated in circulation after gut digestion and absorption is not always related with the chemical form of DHA consumed [115,116,139]. In fact, LC-PUFA are mainly incorporated in maternal plasma PL fraction while saturated and monounsaturated FA in plasma TG [25]. However, several reports in piglets showed higher circulating values of DHA in plasma PL fraction when DHA was administered as PL from different sources: Jiménez et al. in newborn formula-fed piglets after the administration of a formula enriched with LC-PUFA from pig brain concentrate compared to sow milk [111]; Amate et al. after the administration of DHA in the form of PL from egg yolk or TG from fungal and tuna oil [109]; and Alessandri et al. administered DHA-rich egg-yolk vs. fish oil [112]. On the contrary, opposite results with higher DHA enrichment in plasma after DHA-TG ingestion have been reported in rats [106] and no differences in DHA distribution between plasma lipid fractions after the supplementation with PL or TG sources the diet of full-term infants [115]. Vaisman et al. reported higher omega-3 LC-PUFA enrichment in plasma PL compared with placebo, as well as enhanced visual sustained attention score in children, but no differences were observed between PL and TG sources supplementation effects [116].

**Figure 3:**
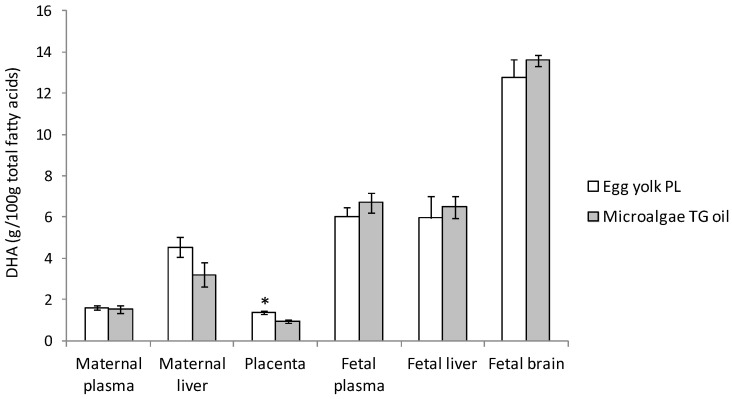
Docosahexaenoic acid (DHA) percentage at delivery in maternal and fetal tissues after maternal DHA supplementation (0.8% of total fatty acids) as phospholipid (PL) from egg yolk or triglycerides (TG) from microalgae oil during the last third of gestation in sows (40 d). Values are means ± SEM (*n* = 6/group). * Indicates significant differences between PL and TG groups of the same tissue (*p* < 0.05). Data from Gázquez et al. 2017 [113].

Studies in pregnant state are scarce. We supplemented the diet of pregnant rats with 2.5% DHA of total FA in the form of PL from egg yolk (mainly PC) or 2.5% DHA in the form of TG from microalgae oil and both sources produced similar values of total DHA in total serum FA and even in plasma PL fraction [107]. Valenzuela and colleagues also reported no differences in the total plasma value of DHA after the administration of this FA as PL or TG in rats at a dose of 8mg/kg/day in adult non-pregnant females, or in these same animals during gestation and after delivery [108]. Nevertheless, they observed a higher value of DHA in the erythrocyte membrane PL after delivery in animals receiving egg yolk PL compared to animals fed with microalgae oil [108]. Our group also carried out a similar experiment with pregnant sows in which animals were fed with diets containing 0.8% DHA from egg yolk (PL form) or microalgae oil (TG form) during the last third of gestation (40 d) (Figure 3) [113]. In this study, despite no differences were observed in total plasma FA profile between groups, we found a non-significant trend towards greater incorporation of DHA in plasma PL fraction in DHA-PL fed group compared to DHA-TG (*p* = 0.130), which indicates that maternal metabolism modulates in certain degree the effect of dietary DHA, modifying its incorporation in maternal serum lipid fractions [113]. The low number of animals per group may have conditioned the lack of statistical significance in DHA PL fraction data (*n* = 6/group). The higher incorporation of DHA was in HDL and LDL lipoproteins [113]. These results of higher DHA in PL fraction after the ingestion of a DHA-PL source obtained in pregnant sows were in line with previous studies performed in non-pregnant animals [109,111,112], probably some inter-species differences in metabolism and in fat sources composition influenced the discrepancies observed between rat and pig studies.

Concerning other maternal tissues (liver, adipose tissue, and brain), the supplementation with preformed DHA as PL or TG contributed equally to DHA levels either in rat or pig models [107,108,113] (Figure 3). However, higher DHA percentage was found in PL, NEFA, and CE fractions of maternal liver after DHA-TG supplementation compared to DHA supplementation from egg yolk DHA-PL in pregnant rats, revealing the high conversion of dietary DHA that takes place also in the liver [107]. Thus, not only digestion and absorption processes but also liver metabolism regulates the incorporation of DHA into different lipid fractions in maternal tissues reducing the impact of the dietary intervention with different fat sources.

We should be cautious comparing studies with different species and under different physiological conditions, since pregnancy is a special situation in which the maternal lipid metabolism is altered in order to provide all the nutrients needed for the developing fetus.

### 4.3. Placental DHA Uptake and Fetal Accretion

The process of placental FA transfer has been extensively reviewed by Larqué et al. [44,102,140]. Placenta can take FA directly from the maternal circulation in the form of NEFA, which concentration increases in the third trimester compared to a non-pregnant woman [31,102]. However, most of the FA present in maternal circulation are esterified in TG, PL, and CE structures. Placenta expresses various lipase activities responsible of the release of FA, in the form of NEFA, from circulating TG and PL, being two of them extensively studied in the literature: lipoprotein lipase (LPL) and endothelial lipase (EL) [141]. LPL preferentially hydrolyzes TG molecules carried in QM and VLDL particles releasing the corresponding NEFA for placental absorption and transfer [142,143]. On the other hand, EL breaks TG but preferably PL [144,145]. EL releases the FA in the sn-1 position of the PL yielding a NEFA and a Lyso-PL with a FA in the sn-2 position [144]. This sn-2 position of PL is usually occupied by LC-PUFA, thus EL may be able to generate DHA-rich Lyso-PL [146].

Once FA have been released, mainly in the form of NEFA, by placental lipases, they enter the cell by passive diffusion or facilitated by protein transports associated with membrane. There are different membrane proteins that have been identified so far: FABPpm, FAT/CD36 and FATP 1–6. Recently, the orphan protein named Major Facilitator Superfamily Domain Containing 2a (MFSD2a) has been described as one of the major carriers of DHA Lyso-PC [147]. When FA are inside the placenta cells, they bind to cytosolic FA binding proteins (FABP). These NEFA can also be oxidized within the trophoblast or reesterified and stored in lipid droplets. Placental cells deliver NEFA to the fetal compartment using the same FA carriers involved in FA uptake from maternal blood [148,149]. A recent study reported higher protein expression of MFSD2a carrier in the basal membrane of the syncitiothrophoblast (in contact to fetal blood) than in the microvillous membrane (in contact to maternal blood), which could indicate that placenta is also able to deliver Lyso-PL to the fetal circulation [150]. Placental metabolism plays an active role in the materno-fetal transfer of FA, combined experimental and computational modeling studies corroborated FA retention and controlled FA delivery to fetal circulation mediated by the placenta [151,152].

To our knowledge, only our research group has evaluated the incorporation of DHA to the placenta and the fetal DHA accretion after maternal supplementation with different DHA fat sources (PL and TG). The administration of 0.8% DHA in the form of PL from egg yolk to pregnant sows produced a significantly higher accumulation of DHA in placenta compared to microalgae DHA-TG (Figure 3) [113]. It may be due to higher release of DHA Lyso-PL from plasma PL lipid pool by the action of endothelial lipase in the placenta [144]. However, Lyso-DHA carrier MFSD2a did not appear to be involved since similar protein expression was observed for DHA-PL and DHA-TG fed groups [113]. This higher accumulation of DHA in placenta was exclusively in PL fraction, which makes sense since placental tissue is composed mainly by PL structures (~85%) [153]. In fact, in vivo [25] and in vitro [154] studies showed that DHA is up-taken by the placenta and esterified mostly as PL and to a lesser extent as TG.

The administration of DHA from PL or TG sources did not produce any differences in placental DHA accumulation in pregnant rats [107]. Again, the inter-species differences seem to affect the results obtained, it is important to mention that the placental structure is not the same in rats and pigs. The placenta of rodents, just like human placenta, is a discoidal endotheliochoreal placenta which represent the minimum separation (one single layer of throphoblast cells) between maternal blood and fetal capillaries while pig placenta is diffuse epitheliochoreal which higher degree of separation between both blood circulations (several layers of epithelial cells). It is unknown whether these histological differences could affect modulate FA uptake or transfer across the placenta.

Despite the discrepancies observed in the placental DHA uptake between different animal models, in all cases the accumulation of DHA in fetal organs (plasma, liver, and brain) was similar regardless of the fat source utilized for maternal DHA supplementation (egg yolk PL or microalgae TG) (Figure 3) [107,113]. Thus, the use of different lipid sources of preformed DHA in the form of PL or TG contributes to a similar fetal DHA accretion, including to the fetal brain. This means that both sources are equally efficient for the fetus.

Nevertheless, Valenzuela et al. showed higher milk DHA content of DHA-PL supplemented rats compared to DHA-TG during the first days of lactation (days 3–20 after delivery), which could have beneficial effects on fetal neurodevelopment [108]. To date no more studies evaluating milk DHA secretion after the use of different DHA sources have been published. It would be interesting to know whether milk DHA content can be increase differentially depending on the lipid source utilized since accelerated fetal brain DHA accretion in human continues up two years of life [2].

A comprehensive understanding of the actual consequences of maternal DHA supplementation during pregnancy for the fetus, especially in the case of fetal brain, is necessary to better design these DHA supplements for pregnant women. Dietary supplements for women need to be tested for real bioavailability and function in the fetus. With the evidence available today, maternal DHA supplementation as PL produces similar materno-fetal transfer of DHA across the placenta and DHA bioavailability for the fetus, including the fetal brain, than DHA in the form of TG. Intestinal digestion and absorption processes, as well as liver FA re-esterification and placental transfer seem to reduce the expected efficiency of different fat sources with DHA. Thus, both sources (PL and TG) are equally available for the developing fetus during pregnancy and can be used for the manufacture of nutritional supplements with DHA for pregnant women.

## 5. DHA Supplementation in Complicated Pregnancies

Some maternal diseases and conditions negatively affect LC-PUFA metabolism and their transfer across the placenta. Tomedi et al. showed that obese pregnant women were 3 times as likely of being in the lowest tertile of essential FA (DHA, EPA, and AA) [155]. Consistent with this, positive associations between maternal body mass index (BMI) and mid-pregnancy AA and omega-6 PUFA while negative association with total omega-3 PUFA has been observed in the Dutch Generation R cohort [156]. Moreover, several studies have shown reduced plasma values of DHA and AA in fetuses of mothers affected by type I diabetes [157], type II [158] and gestational diabetes mellitus (GDM) [159,160].

Our group demonstrated impaired materno-fetal transfer of DHA in GDM pregnancies by the administration of stable isotopes labeled-FA to pregnant women while the transfer of the rest of studied FA was increased [160]. Placenta of these GDM subjects showed lower expression of MFSD2a transporter compared to healthy women and this is associated with a lower DHA concentration in cord blood, which supports the contribution of Lyso-PL to materno-fetal DHA transport [161]. These results were corroborated by Soygur et al. [162], which reinforces the role that MFSD2a protein may play a role in the selective transfer of DHA not only in the brain but also in the placenta. GDM also produces changes in the expression of other FA transport proteins (e.g., FAT, adipocyte FA binding protein (A-FABP) and FATP-1) and activates the insulin signaling cascade which may promote greater transport of fat to fetus [163].

An increase in mRNA and expression levels of lipoprotein receptors in placenta (LDL and VLDL receptors) has been reported in women with GDM depending on their BMI [164]. Obese placentas has shown decreased expression of FATP-4 and increased expression of FAT which could be affecting the FA uptake and transfer process [165]. Besides, an inverse association was found between DHA level in cord blood and pre-pregnancy BMI of mothers [166]. Interestingly, we found in obese pregnant women that big placentas had lower levels of PC carrying DHA and AA in intracellular lipid depots or lipid droplets, indicating not only structural alterations but also changes in FA metabolism [167]. Nevertheless, more studies and with more subjects are needed to establish the mechanisms underlying these alterations observed in GDM or obese placentas.

Some recent studies suggest lower efficiency of dietary DHA supplementation in obese and GDM pregnancies [168,169]. Monthe-Dreze et al. showed that, despite all BMI groups of pregnant women had higher omega-3 concentrations after supplementation, obese women had attenuated changes compared to lean women, resulting in a 50% difference in the effect size [169]. Min et al. supplemented GDM women with 600mg/d DHA or high oleic acid sunflower seed oil and they observed enhanced maternal but not fetal DHA status, which may indicate that placenta lowered the effect of DHA supplementation [168].

To our knowledge, no studies evaluating the effect of different DHA sources or structures (e.g., PL vs. TG) have been conducted in complicated pregnancies. All this together with the high prevalence of diabetes and obesity increases the need for new sources with greater bioavailability of DHA for the placenta and, more importantly, for the fetus.

## 6. Conclusions

The animal model used to evaluate the materno-fetal transfer of DHA as PL or TG is a key issue since placental structure differs among the species used for such studies. Administration of DHA-rich PL produces a modest enrichment of DHA in PL plasma lipid fraction in piglets and pregnant sows compared to DHA-TG administration while similar or opposite results have been observed in other species. Intestinal digestion, re-esterification in both gut enterocytes and liver, as well as placental transfer processes reduce the impact of the dietary intervention with different lipid sources on fetal DHA levels. Dietary lipid utilization and bioavailability comprises several metabolic processes that are not completely understood and further research is needed. There are a limited number of studies evaluating placental and fetal accretion of DHA after the administration of different fat sources to pregnant animals (PL and TG). Despite some differences observed in placental DHA content between animal species, fetal DHA accretion and, especially, fetal brain DHA accumulation after PL or TG administration was similar. However, it is important to note that the use of animal models (rodents and pigs) in most studies might have some limitations in extrapolating results to humans. Lyso-PL have been proposed as a preferred physiological carrier of DHA to the brain, the available data on DHA Lyso-PL bioavailability with respect to other sources are promising and seem to indicate an increased DHA incorporation in some tissues but more studies are needed to evaluate their effects during pregnancy, fetal bioavailability, and long-term effects on neurodevelopment. In summary, although most of the results available were obtained in animal models, both PL and TG sources can be used for the manufacture of DHA supplements during pregnancy since they show a comparable bioavailability and promote similar DHA accretion in the fetus. The dose of DHA administered is perhaps more decisive than the fat source to increase fetal DHA status.

## Figures and Tables

**Table 1:** Studies evaluating docosahexaenoic acid (DHA) bioavailability using Lyso-Phospholipids (Lyso-PL) respect to other chemical forms.

Ref.	Age	Pregnant	DHA Sources	DHA Dose	Mode of Administration	Outcomes Measured	Major Findings
Rat/mouse models							
[79]	Young male rats	No	^3^H-DHA as Lyso-phosphatidylcholine (Lyso-PC) and NEFA	12 nmol	Tracer infusion	^3^H-DHA enrichment in brain, liver, kidney and heart	↑ Incorporation of ^3^H-DHA as Lyso-PC in the brain. Similar or ↓ incorporation in other tissues compared to NEFA
[81]	Adult male mice	No	Lyso-PC and NEFA	40 mg/kg/d	Oral intake (30 days)	Plasma, liver, adipose and different brain regions fatty acids (FA). Brain function and memory tests	Lyso-PC but not NEFA increase brain DHA content. No differences in other tissues. ↑ Improvement of brain function and memory with Lyso-PC
[85]	Adult female rats	Yes	Monoacylglycerol and Lyso-PC	8 mg/kg/d	Maternal supplementation (9 weeks)	Blood, liver and adipose tissue FA in mothers. Brain regions FA in the offspring. Learning and memory skills	↑ Incorporation of DHA in cerebellum and hippocampus of pups with Lyso-PC DHA while no differences in frontal and occipital cortex. Better learning and memory scores in Lyso-PC offspring
[86]	Young male rats	No	^14^C-DHA as Lyso-PC and NEFA	100 nmol	Tracer infusion	^14^C-DHA enrichment in plasma, brain, heart, eyes and liver FA	↑ ^14^C-DHA incorporation in brain after Lyso-PC administration. No differences in other tissues
[87]	Old male rats	No	^14^C-DHA as Lyso-PC and NEFA	10 μCi	Tracer infusion	^14^C-DHA enrichment in plasma and different brain PL pools	↓ Net rate of DHA entry into the brain with Lyso-PC ↑ ^14^C-DHA incorporation in brain PC but ↓ in ethanolamine PL with Lyso-PC
[88]	Adult male rats	No	Lyso-PC, PL and TG	40 mg/kg/d	Oral intake (30 days)	Plasma, liver, heart, adipose tissue and different brain regions FA	Incorporation of DHA in plasma and liver: Lyso-PC > PL > TG. ↑ Incorporation of DHA from TG in heart and adipose tissue. Incorporation of DHA in brain regions: Lyso-PC > PL while no effect of DHA TG
[89]	Adult male rats	No	Lyso-PL and TG	23.5 mmol/kg diet	Oral intake (28 days)	Serum and liver FA	No differences of DHA incorporation in serum. ↑ Incorporation of DHA from TG in liver
Human studies							
[90]	Adult men	No	^13^C-DHA as Lyso-PC and in the form of TG	50 mg	Single oral intake	^13^C-DHA enrichment in plasma and red blood cells PL FA	↑ ^13^C-DHA incorporation in plasma PL with Lyso-PC. No differences in red blood cells PL

DHA; docosahexaenoic acid; FA, fatty acids; Lyso-PC, lyso-phosphatidylcholine; NEFA, non-esterified fatty acid; PL, phospholipids; TG, triglycerides. ↑ increase, ↓ decrease.

**Table 2:** Studies evaluating docosahexaenoic acid (DHA) bioavailability using triglyceride (TG) respect to phospholipid (PL) sources.

Ref.	Age	Pregnant	DHA Sources	DHA Dose	Time of Administration	Outcomes Measured	Major Findings
Rat models							
[104]	Adult female	No	Fish oil (TG) and krill oil (PL)	1.9–4.6%	8 weeks	FA apparent digestibility and brain fatty acids (FA)	↓ Intestinal absorption and brain DHA deposition after administration as PL
[105]	Adult female	No	Tuna/fungal oil (TG) and pig brain concentrate (PL)	0.9%	3 weeks	FA excretions and fat apparent absorption	↓ Apparent absorption of DHA from pig brain PL
[105]	Adult female	No	Egg TG and egg PL	0.9%	3 weeks	FA excretions and fat apparent absorption	↑ Apparent absorption of DHA from egg PL
[106]	Adult male	No	TG and PL oils (not specified)	~1%	3 weeks	Plasma, liver and kidney FA	↓ DHA in plasma and liver after PL oil administration
[107]	Adult female	Yes	Microalgae oil (TG) and egg yolk (PL)	2.5%	3 weeks	Maternal plasma and liver FA, total fetus and fetal brain FA, placenta FA	No DHA differences in maternal plasma, fetus or placenta. ↑ DHA in maternal liver fractions with TG source
[108]	Adult female	Yes	Microalgae oil (TG), egg yolk (PL)	8 mg/kg/d	9 weeks	Maternal plasma, red blood cells, liver, adipose tissue and milk FA	No differences in maternal plasma. ↑ DHA in red blood cells and milk FA with PL source
Pig models							
[109]	Piglets	No	Tuna/fungal oil (TG) and egg yolk (PL)	0.3%	4 weeks	Plasma and plasma lipoprotein lipid fractions FA	↑ DHA incorporation in HDL-PL fraction with egg yolk source (PL)
[110]	Piglets	No	Tuna/fungal oil (TG) and egg yolk (PL)	0.3%	16 days	Plasma FA and dry matter digestibility	↓ Intestinal absorption and plasma concentration of DHA after administration as PL
[111]	Piglets	No	Sow milk (TG) and pig brain concentrate (PL)	0.3–0.4%	17 days	Plasma PL and liver microsomes FA	↑ DHA incorporation in plasma PL and liver with DHA-PL source
[112]	Piglets	No	Fish oil (TG) and egg yolk (PL)	0.2–0.4%	2 weeks	Plasma and red blood cells FA	↑ DHA incorporation in plasma PL and with DHA-PL source
[113]	Adult female	Yes	Microalgae oil (TG) and egg yolk (PL)	0.8%	6 weeks	Maternal plasma, lipoproteins and liver FA, fetal plasma and brain FA, placenta FA	↑ DHA content in placenta with PL source but no differences in fetal tissues
Human studies							
[114]	Preterm infants	No	Breast milk/algae oil (TG) and egg yolk (PL)	0.24–0.64%	≥5 weeks	Fecal output and FA balance	↑ Intestinal absorption of DHA administered as PL
[115]	Full term infants	No	Microalgae oil (TG) and egg yolk (PL)	0.1%	3 months	Plasma lipid fractions FA	No differences in plasma DHA
[116]	Children 8–13 y	No	Fish oil (TG) and enriched PL (not specified)	100 mg/d	3 months	Plasma and red blood cells PL fraction FA	No differences in plasma or red blood cells DHA

DHA; docosahexaenoic acid; FA, fatty acids; PL, phospholipids; TG, triglycerides. ↑ increase, ↓ decrease.

## Data Availability

No new data were created or analyzed in this study. Data sharing is not applicable to this article.
